# Activation of DNA Damage Response Induced by the Kaposi’s Sarcoma-Associated Herpes Virus

**DOI:** 10.3390/ijms17060854

**Published:** 2016-06-01

**Authors:** Enea Gino Di Domenico, Luigi Toma, Valentina Bordignon, Elisabetta Trento, Giovanna D’Agosto, Paola Cordiali-Fei, Fabrizio Ensoli

**Affiliations:** 1 Clinical Pathology and Microbiology Department, San Gallicano Institute, IRCCS, Rome 00144, Italy; bordignon@ifo.it (V.B.); trento@ifo.it (E.T.); dagosto@ifo.it (G.D.); cordiali-fei@ifo.it (P.C.-F.); ensoli@ifo.it (F.E.); 2 Infectious Disease Consultant, San Gallicano Institute, IRCCS, Rome 00144, Italy; toma@ifo.it

**Keywords:** DNA damage response, kaposi, sarcoma, tumor, skin, cancer

## Abstract

The human herpes virus 8 (HHV-8), also known as Kaposi sarcoma-associated herpes virus (KSHV), can infect endothelial cells often leading to cell transformation and to the development of tumors, namely Kaposi’s sarcoma (KS), primary effusion lymphoma (PEL), and the plasmablastic variant of multicentric Castleman’s disease. KSHV is prevalent in areas such as sub-Saharan Africa and the Mediterranean region presenting distinct genotypes, which appear to be associated with differences in disease manifestation, according to geographical areas. In infected cells, KSHV persists in a latent episomal form. However, in a limited number of cells, it undergoes spontaneous lytic reactivation to ensure the production of new virions. During both the latent and the lytic cycle, KSHV is programmed to express genes which selectively modulate the DNA damage response (DDR) through the activation of the ataxia telangiectasia mutated (ATM) pathway and by phosphorylating factors associated with the DDR, including the major tumor suppressor protein p53 tumor suppressor p53. This review will focus on the interplay between the KSHV and the DDR response pathway throughout the viral lifecycle, exploring the putative molecular mechanism/s that may contribute to malignant transformation of host cells.

## 1. Introduction

Viruses are involved in the pathogenesis of various human tumors. It has been reported that approximately 11% of human cancers can be attributed, either directly or indirectly, to infecting viruses [1]. These include the human papillomavirus (HPV), the hepatitis B and C viruses (HBV and HCV, respectively), the Epstein-Barr virus (EBV), the Human immunodeficiency virus (HIV), and the Kaposi’s sarcoma-associated herpes virus (KSHV) [2]. In fact, KSHV has been classified as a group 1 biologic carcinogenic agent by the International Agency for Research on Cancer due to its ability to induce the development of Kaposi’s sarcoma (KS) as well as of two rare lymphoproliferative diseases, namely primary effusion lymphoma (PEL) and the plasmablastic variant of multicentric Castleman’s disease, both arising from infection of B cells [3]. KSHV is not ubiquitous and large geographic variations in prevalence can be observed. The highest rates of prevalence are reported in sub-Saharan Africa (>50%), moderate rates in Mediterranean countries, (20%–30%) and low rates in Europe, the United States and Asia (<10%) [4,5,6].

Extensive epidemiologic studies confirmed the presence of KSHV genome in all the KS variants, demonstrating a strong temporal association, which indicates KSHV as the necessary agent for KS development. However, since only a small percentage of KSHV-infected individuals develop KS, it is clear that other factors, in addition to KSVH infection, are required for tumor development and progression. The pathogenic processes leading to KS development appear strongly associated with KSHV reactivation from latency [7]. This event represents a critical pathogenic phase that induces the production of viral factors interfering with host cellular activities, allowing the virus to evade the host immune response [8]. In addition, host predisposing factors, including either immune suppression or an unbalanced immune response, appear to play a relevant role in promoting the disease. In fact, either an association with HIV infection [9,10] or co-infections with other viruses (EBV, HHV-6) or bacteria as well as the presence of a predisposing genetic background [11,12,13], appear to be necessary for the development and progression of KSHV-associated diseases [14].

On the other hand, it is now clear that multiple KSHV genes, which regulate the virus life cycle controlling the latent and lytic replication, also have oncogenic potential by acting on the cellular DNA damage response pathways [15].

## 2. Role of the DNA Damage Response (DDR) Pathway in KSHV Life Cycle

The cellular response to DNA damage comprises different surveillance mechanisms that guarantee genome stability and cell survival. The production of simultaneous breakage of the two complementary DNA strands induces activation of double-strand break (DSB) repair mechanisms, delay or arrest of cell-cycle progression, and eventually programmed cell death [16]. This mechanism has been shaped through evolution in order to prevent losing genetic information and to protect the genome from DSBs. Damaged DNA triggers a response that mainly relies on the phosphatidylinositol 3-kinase-like protein kinase (PIKK), members of serine/threonine kinases group, which include ataxia telangiectasia mutated (ATM), ataxia telangiectasia and Rad3 related (ATR), and DNA-dependent protein kinase catalytic subunit (DNA-PKcs). Collectively, by the phosphorylation of a large number of downstream elements, the PIKKs factors, cooperate in the recruitment of repair factors or, in case of irreparable damage, to progress the cells through senescence and/or apoptosis [17,18].

Two major pathways are involved in DSBs repair: non-homologous end-joining (NHEJ) and homologous recombination (HR). NHEJ is active throughout the cell cycle and relegates broken ends without the need of extensive processing [19]. NHEJ is efficient in repairing damage, but occasionally it can induce mutations at the joining sites. Conversely, HR is an error-free process that requires the presence of long and undamaged 3’-single-strand (ss)DNA of homologous DNA template during the S/G2 phase, to repair the broken ends [20]. By controlling DDR signaling, ATM and ATR are also master regulators of HR and NHEJ. In particular, ATM is activated by DSBs, while ATR is activated at regions exposing ssDNA [19,20].

After initially “sensing” DNA damage, ATM kinase is converted into a partially active monomer by an autophosphorylation process on S1981 and its interaction with MRN at the DSB site [21]. Complete activation of ATM is further achieved through lysine acetylation by Tip60 [22]. Once ATM is activated by MRN, its phosphorylation level oscillates during the repair process, due to the activity of different phosphatases [23]. Efficient DSB repair requires chromatin remodeling and this process is triggered by ATM-dependent phosphorylation on S139 of the histone H2A variant (γ-H2AX) [24]. This histone modification spreads over about 2Mb surrounding a break [25,26]. Chromatin relaxation, in the area surrounding DNA damage, contributes to the process by potentiating ATM signaling [27,28]. These initial events promote the recruitment of other DNA repair factors at sites of damage that induce the phosphorylation of downstream targets of ATM such as CDC25, CHK2, and p53 modulating cell cycle arrest and apoptosis [17,23].

As previously mentioned, ATR is specifically activated at single-strand DNA regions, at the site of ssDNA-dsDNA junctions, via a process that involves ATR-interacting protein (ATRIP), and replication protein A (RPA), which coats the exposure of long stretched ssDNA [29]. In addition, the heterotrimeric checkpoint clamp 9-1-1, composed by RAD9-RAD1-HUS1, recruits the topoisomerase-binding protein 1 (TopBP1) to the sites of damage. Recruitment of the checkpoint clamp 9-1-1 stimulates ATR kinase activity, leading to the phosphorylation of downstream effectors [30].

DNA-PKcs is the central regulator of the NHEJ repair, operating together with the KU heterodimer as a DNA end-bridging factor and in association with the MRN complex, which tethers the DNA ends of DSB together [31]. ATM and ATR also phosphorylate downstream kinases. CHK1 is the primary effector of the intra-S and G2-M checkpoints, whereas CHK2 participates in the G1-S and intra-S checkpoints [32]. CHK1 and CHK2, specifically phosphorylate CDC25 phosphatases, which, in turn, suppress cyclin-dependent kinase 2 (CDK2) activity inducing cell-cycle arrest [17]. Moreover, both CHK1 and CHK2 can phosphorylate p53, affecting cell cycle progression and apoptosis. In unstressed cells, p53 is a short-lived protein and its degradation is promoted by the mouse double minute (MDM2) gene [17]. After DNA damage, ATM and CHK2 phosphorylate p53 (S15 and S20), thus reducing its ability to bind MDM2 and contributing to its stabilization [33,34]. ATM can also directly phosphorylate MDM2 at S395, leading to a reduction in MDM2 activity [35]. Additionally, the CHK1 and CHK2 kinases participate in DNA repair through the phosphorylation of elements involved in the homologous recombination pathway [35].

The mammalian DNA repair machinery has also evolved mechanisms to recognize the presence of viral genetic material [36]. Nevertheless, it is conceivable that viral DNA might be recognized as damaged DNA, and this may trigger the DDR thus endangering an effective viral replication. To avoid this, viruses have also developed a sophisticated mechanism to engage and subvert the DDR signaling pathway to protect and promote self-propagation [36,37].

Like all herpes viruses, the KSHV lifecycle consists of latent and lytic replication phases, respectively [38]. During latent infection, the KSHV genome persists in the nucleus of the host cell as a circular double-stranded DNA molecule [39,40]. In this phase, a very limited array of viral genes is expressed. Their function is to ensure the silencing of viral lytic genes, allowing the survival and proliferation of infected cells as well as the maintenance, replication, and segregation of the viral episomes into daughter cells during mitosis. 

Thus, KSHV latently infected cells constitute a virus reservoir which chronically persist in the host, while virus overt replication is tightly controlled by the host immune system. Establishing and maintaining latency involves a limited number of viral and cellular factors. The limited array of viral genes expressed during latency include latency-associated nuclear antigen (LANA), viral cyclin (v-Cyclin), viral Fas-associated death domain-like interleukin-1β-converting enzyme-inhibitory protein inhibitory protein (v-FLIP), the kaposins, and also 12 miRNAs located in the latency locus of the KSHV and antisense RNAs [41]. By silencing KSHV lytic gene expression, these genes play key roles in escaping the host immune surveillance, thus ensuring the establishment of a lifelong persistent infection [41]. On the other hand, latent infection appears to have a critical role in the development of KSHV-associated malignant transformation, since it is capable of promoting cell growth and survival. In fact, LANA is strongly expressed in all infected cells and in all KSHV-positive KS tumors [42]. LANA promotes latency through an active transcription repression of several lytic genes [41]. Moreover, by interacting with the H2AX histone, LANA promotes the tethering of the viral episome to host DNA, ensuring its distribution to daughter cells during mitosis [43,44,45]. LANA has been also directly implicated in tumorigenesis through the inhibition of the host tumor suppressor pathways controlled by p53 and Rb-E2F [41,46,47,48] and by activating telomerase reverse transcriptase (TERT) expression that, in turn, acts in promoting infected-cell survival [41,49].

KSHV v-Cyclin, homologue of the mammalian D-type cyclin counterparts, acts as a constitutive activator of cellular cyclin-dependent kinase 6 (CDK6) [50]. The v-Cyclin/CDK6 kinase complex regulates cell cycle progression as well as virus replication [51,52,53,54].

Viral FLIP (vFLIP), homologous to the cellular FLIPs, inhibits autophagy in KSHV-infected endothelial cells and lymphocytes [55] and can activate anti-apoptotic genes [41,55,56], thus inhibiting cell senescence [57]. The Kaposins, encoded by K12 are three proteins namely, Kaposin A, B, and C, expressed in latent infection but also present during lytic reactivation. Kaposins have the ability to drive cell transformation contributing to the inflammatory mileau present in KS. In particular, Kaposin B is able to promote the upregulation of inflammatory cytokine levels through the activation of the p38 and mitogen-activated protein kinase-activated protein (MAPKAP) kinase 2 (MK2) [58,59]. Moreover, KSHV expresses approximately 20 viral microRNAs (miRNAs) originating from 12 stem-loop precursors. These noncoding RNAs participate in establishing and maintaining KSHV latent infection *in vivo* and in oncogenesis by miRNA-mediated RNA interference of host cell mRNAs [60,61]. Most KSHV miRNAs are expressed during latency, but miRNA K10 and -K12 are further expressed upon viral reactivation [62,63,64], suggesting also a putative role in lytic infection.

The switch from KSHV latent to lytic replication can be triggered by specific intracellular signals or extracellular stimuli in a small proportion of cells, including hypoxia, HIV infection, inflammatory cytokines, and chemical agents, such as histone deacetylase (HDAC) inhibitors or protein kinase C agonists [65,66]. Lytic replication triggers the expression of more than 80 transcripts, releasing infectious virions and cell lysis [67]. Importantly, virus lytic replication allows the dissemination of the virus from the latent reservoir of infected cells, inducing tumor development. Infected endothelial cells in the KS tumor show typical spindle morphology with the majority of cells latently infected and few undergoing lytic replication [68]. In contrast to latency, the lytic replication program involves a large set of viral genes. The expression of these genes is tightly regulated and time-controlled. In fact, lytic genes can be grouped, according to their timing of expression, as immediate early (IE), early (E), and late (L) genes [69]. Expression of the viral regulator of transcription activation (RTA) protein, which is a potent transcriptional activator with sequence-specific DNA binding potential, initiates lytic replication acting both in primary infection and during reactivation [40,70]. Importantly, several factors involved in both latent and lytic genetic programs are implicated in the regulation of the DDR pathway, contributing to genomic instability and ultimately to promoting the development of KSHV-related malignancies [37].

## 3. Activation of the DDR during KSHV Latency

Most of the tumor cells in KS, primary effusion lymphoma (PEL) and multi-centric Castleman’s disease are latently infected by KSHV [71,72,73]. During latency the virus maintains an episomal structure expressing only a subset of viral transcripts. This limited number of genes includes: LANA encoded by ORF73, the viral cyclin D (v-Cyclin) encoded by ORF72, vFLIP encoded by ORF71, and K12/Kaposin family of proteins (Kaposin A, B, and C) as well as 12 miRNAs that can be further processed to yield 18 miRNAs [41,42]. The transcriptional organization of the major latency genes LANA, v-Cyclin, and v-FLIP indicates that they are closely located and their transcription is regulated by a constitutively active promoter (LT_c_) located upstream from LANA [42]. The K12 gene is transcribed from a separate promoter, the kaposin promoter (LT_d_), located downstream from LANA. The LT_d_ promoter encodes the kaposin proteins and a bicistronic RNA for v-Cyclin and v-FLIP [50]. This promoter also controls the expression of all KSHV miRNAs, which are encoded on the same strand and constitutively expressed in latently infected PEL cells [50]. The ORF74-K14 transcript has also been detected in some KS lesions and it can be co-expressed together with LANA–v-Cyclin–vFLIP from the same locus in latently infected cells [74].

KSHV can thus establish and maintain latent infection while promoting cell proliferation and preventing apoptosis. This is associated with elevated levels of DNA damage and chromosomal aberrations due to KSHV exploitation of the host cell DDR machinery. The engagement of the DDR appears to be key in promoting viral persistence while avoiding cell cycle arrest or apoptosis that, in turn, would negatively impact the viral lifecycle [37]. In particular, several molecules involved in the DDR pathway are downregulated at the transcriptional level throughout KSHV infection [41]. These include ATM, ATR, DNA-PKcs, MRN, and the KU70/86 complex, which comprise different surveillance mechanisms that guarantee genome stability and cell survival in response to DNA damage [75]. Proteins such as H2AX, BRCA1, 53BP1, and MDC1, involved in the DNA damage cascade, are target substrates for ATM phosphorylation as well as the checkpoint proteins CHK1 and CHK2 that can phosphorylate p53, with downstream consequences on cell cycle progression and apoptosis [18]. The ability to regulate the DDR response and promote viral replication is a well-known strategy adopted by adenovirus and by other human viruses like, HPV, EBV, HBV HCV human T-cell leukemia virus type 1 (HTLV-1), and Merkel cell polyomavirus (MCPyV) [36,37]. The possibility to modulate the DDR genes may also represent a key feature in suppressing apoptosis signals induced by KSHV infection or regulating the cell cycle checkpoints thus promoting viral replication [37].

**Figure 1:**
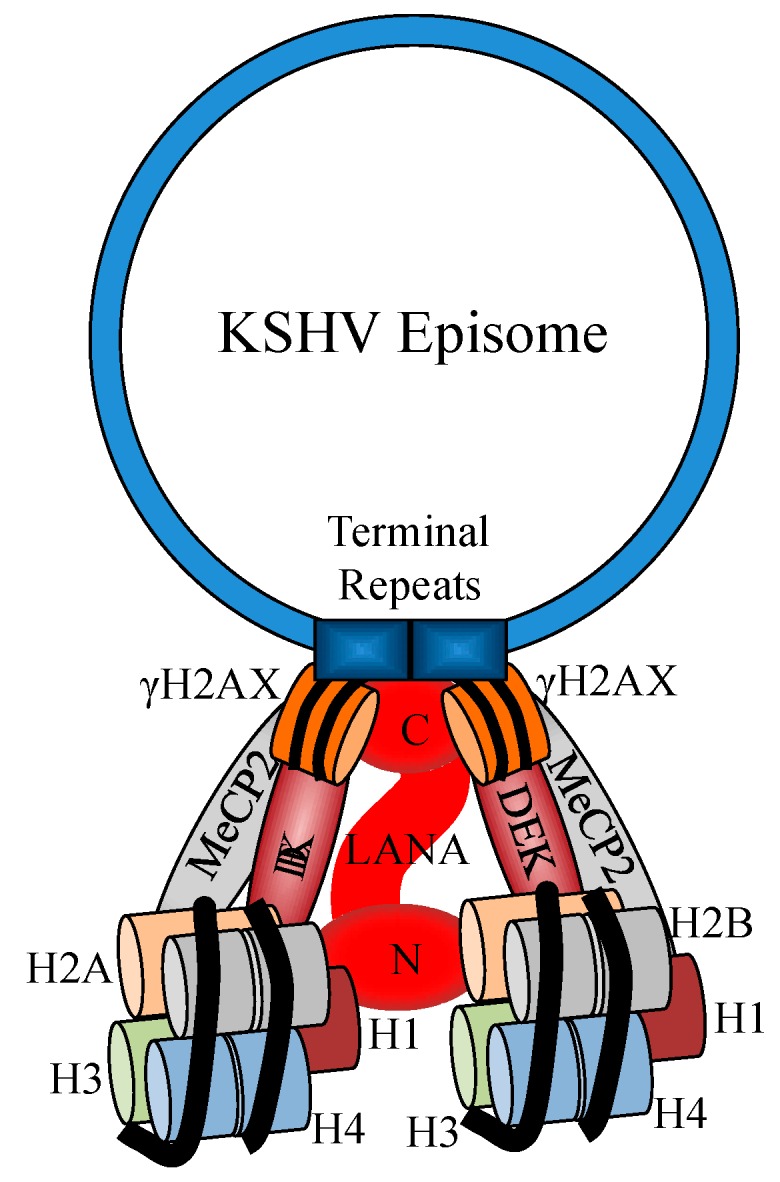
Schematic representation of latency-associated nuclear antigen (LANA)-mediated Kaposi sarcoma-associated herpes virus (KSHV) episomal persistence after infection. LANA binds to LANA binding sequences in the episome terminal repeats by its C terminal region. In this model, γH2AX participates in KHSV episomal persistence possibly by strengthening the interaction between C-LANA and terminal repeat (TR) as proposed by Jha *et al.*, 2013 [45].

All latently infected cells and KSHV-positive KS tumors express the multifunctional viral protein LANA. Among the group of latent proteins, LANA is the most frequently detected antigen in KSHV-infected cells of KS, PELs, and MCD and it is considered a hallmark of KSHV genome persistence [76]. LANA is a large (220–230 kDa) nuclear protein [77] required for maintaining episomic viral forms by supporting KSHV latent DNA replication and tethering the viral episome to cellular chromosomes [41]. In fact, depletion of LANA is accompanied by a strong reduction of viral episomes in infected cells and a lack of virus latency [67]. This suggests that the presence of LANA in the host cells is required for long-term persistence of the viral DNA. LANA tethers the circular viral DNA to the host chromosomes. This is a key mechanism required for redistributing the episomic KSHV genome to daughter cells during cell division (Figure 1) [67,78,79]. LANA possesses an amino-terminal domain with a chromatin binding sequence (5–22 aa) that binds the host chromatin at nucleosomal level through a multiprotein complex including histones on the cellular chromatin [79,80,81,82]. In addition, several host molecules associated with LANA cooperate in episome binding. These proteins include histones H1, H2A, H2B, Brd2 Ring3, Brd4, DEK, MeCP2, NuMA, CENP-F, and Bub1 [44,45]. Episome binding of LANA results from the direct association of LANA with a large DNA-protein complex within the KSHV terminal repeats (TRs) [45,80,83,84].

Immunofluorescence and immuno-FISH analysis revealed that LANA co-localizes with the phosphorylated form of the histone H2AX (γH2AX). Furthermore, chromatin immunoprecipitation assays revealed that a reduction in H2AX levels limits the binding of LANA with KSHV terminal repeats (TRs) [45]. H2AX is a variant of histone H2A, a central element in the DDR pathway [85,86]. H2AX possesses high sequence homology with H2A, representing approximately 10% to 15% of total cellular H2A [87]. The histone H2AX is the DDR molecule found to be up-regulated in the presence of KSHV infection. H2AX is considered to be a critical molecule in the DDR signaling pathway, and its phosphorylation is one of the hallmarks of DNA damage [87]. The binding of LANA to H2AX contributes to its phosphorylation, functioning as a molecular signal for TR identification [45]. The observation that both N and C terminus residues of LANA are able to bind γH2AX suggests that γH2AX contributes to the binding of LANA to the TRs [45]. LANA anchors to the host chromatin in association with N terminus binding proteins (e.g., H2A, H2B, H1, Bub1, CENP-F) and C terminus binding proteins (e.g., DEK, NuMA, Bub1, MeCP2, CENP-F) [44,45,79,88,89].

**Figure 2:**
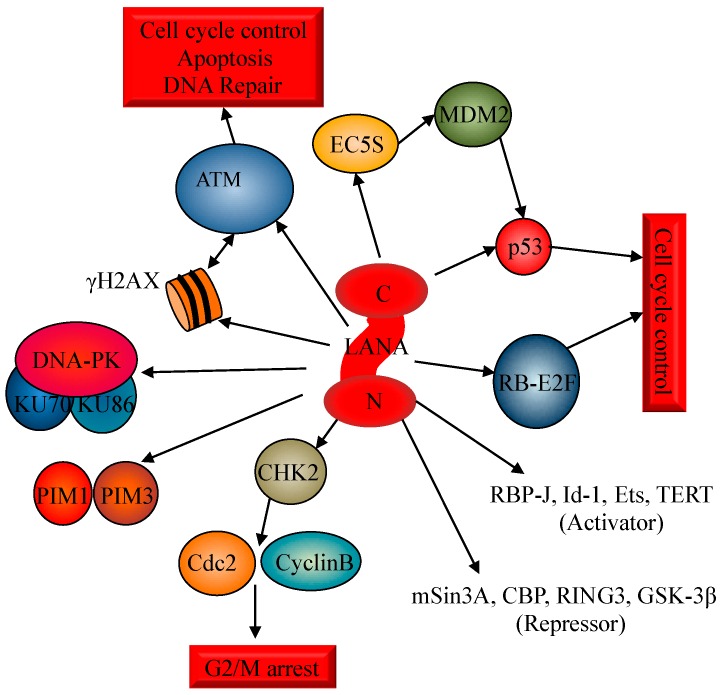
LANA and the interacting factors involved in the DNA Damage induction as reported in the text.

KSHV infection of primary endothelial cells induces phosphorylation of H2AX and ATM as early as 30 min post-infection and persists during virus latency [90]. The elevated levels of γH2AX that were observed during KSHV infection were attributed to a decrease in the degradation of H2AX Lys48-linked polyubiquitination and to a corresponding increase in Lys63-linked polyubiquitination, which lead to promote protein stability [90]. Inhibition of ATM kinase activity and H2AX knockdown reduced the expression of the LANA gene. Moreover, knockdown of H2AX caused a reduction of more than 80% of the nuclear KSHV DNA copy numbers impairing the ability of the virus to establish latency. Comparable results were also obtained in ATM-negative cells demonstrating that both ATM and H2AX play important roles in establishing KSHV latency [45]. These results suggested that KSHV regulates activation of the ATM-dependent DDR pathway (Figure 2) while inhibiting downstream signaling to favor its survival advantage [90]. Moreover, LANA is also involved in the disruption of the cyclin B and CDC2 mediated G2/M checkpoint response by directly binding to the serine rich domain of the amino-terminal region of CHK2 within the nucleus of B-cells, therefore promoting the release of nocodazole induced G2/M arrest [91]. Thus, LANA triggers the release of the G2/M checkpoint arrest by interacting with CHK2 and therefore modulating the ATM/ATR signaling pathway [91].

Defects in these checkpoints are repeatedly observed in cancer cells and in cells infected with DNA transforming viruses, such as adenovirus, simian virus 40, papillomavirus, and Epstein Barr virus. Therefore, alterations of the cell cycle regulation pathways may promote tumorigenesis while the ATM/ATR regulated checkpoint acts as a guard against tumor progression [91]. LANA can further contribute to tumorigenesis by interacting with several transcription factors. Indeed, LANA induces the transcriptional activation of different anti-tumorigenic pathways such as RBP-J, Id-1, Ets and the human telomerase reverse transcriptase (TERT) promoter through interaction with the transcription factor Sp1 [89,92,93,94]. On the other hand, LANA represses mSin3A, CBP, RING3, glycogen synthase kinase 3 (GSK-3β) [46,81,95,96] and also inhibits the activities of the p53 [46,48] and the RB–E2F tumor suppressor pathways [47], two other important targets of ATM [18,97]. 

In particular, LANA operates as a component of the EC5S ubiquitin complex targeting p53 for degradation [66]. Moreover, LANA is able to bind to both p53 and MDM2 proteins. The use of the MDM2 inhibitor Nutlin-3a disrupts the p53-MDM2-LANA complex inducing a specific and highly potent activation of apoptosis in PEL cells [98]. Also, LANA interacts with several kinases including GSK-3 [99,100], PIM-1, and PIM-3 [101], and the N-terminal region of LANA interacts with the catalytic subunit of DNA-PKcs, KU70, and KU86 [102]. Finally, LANA is also phosphorylated by an unidentified kinase mediated through an interaction of LANA with RING3 [103].

v-Cyclin has been demonstrated to be present in most of the malignant cells associated with KSHV infection, suggesting that this protein can act as a putative viral oncogene [52]. The ability of tumor viruses to modulate the cell cycle allows for the establishment of a constitutive S phase-like environment necessary to support viral replication [104]. The result of this constitutive S-phase induction is the inappropriate entry into mitosis, which consequently causes DNA damage and DDR checkpoint activation [105,106]. The expression of KSHV v-Cyclin in telomere-immortalized human endothelial cells leads to the activation of DDR markers, as shown by the phosphorylation of ATM signaling pathway, H2AX, CHK2, and p53 and S-phase arrest [107]. v-Cyclin is the viral homologue of cellular cyclin D. The sequence of the v-Cyclin shows approximately 53% sequence similarity to cyclin D2, with high homology in the cyclin box [108]. Like its cellular homologue, v-Cyclin binds and constitutively activates the cyclin-dependent kinase 6 (CDK6) [108,109]. Depletion of CDK6 showed a significant reduction of ATM and CHK2 phosphorylation, suggesting that v-Cyclin–induced DNA damage checkpoint is dependent on CDK6 expression. Conversely, depletion of CDK4 or CDK2 did not affect the DNA damage response, indicating that DNA damage-induced checkpoints caused by v-Cyclin is not dependent on these cyclins [107]. The v-Cyclin–CDK6 complex, like its cellular counterpart, regulates cell cycle and cell proliferation and has the ability to phosphorylate pRb, promoting accelerated S-phase entry. These functions reveal that v-Cyclin resembles D type cyclins both at the structural and functional levels [52,109]. Nevertheless, the v-Cyclin–CDK6 complex shows enhanced kinase activity towards their substrates, as measured by *in vitro* kinase assays, and a broader substrate range than the cellular D type cyclin–CDK4/6 complexes [52,110]. The v-Cyclin–CDK6 may produce a more stable active complex with increased kinase activity which mediates phosphorylation and inhibition of its cellular target protein pRb, Histones H1, cyclin-dependent kinase inhibitor 1 p27/Kip1, p21/Cip1, and p21/Cip1 [42,111]. 

v-Cyclin expression in primary cells revealed a p53-dependent growth arrest and cytokinesis defects. These restrictions were overcome by the loss of p53, pointing to the oncogenic potential of v-Cyclin [112]. Moreover, p53 is strongly activated in cells expressing v-Cyclin, and its activity appears to be necessary for the inducing oncogene-induced senescence through the up-regulation of the autophagy regulators Sestrin1 and damage-regulated autophagy modulator (DRAM). Notably, oncogene-induced senescence is associated with mTOR pathway inactivation, partially dependent on AMP-responsive protein kinase (AMPK), a target of Sestrin1 [113]. Moreover, KSHV v-Cyclin expression promotes cytokinesis defects, amplification of centrosomes, polyploidy and intra-S phase growth arrest [107,112]. 

The v-Cyclin/CDK6 kinase also mediates phosphorylation of nucleophosmin (NPM) an indirect target of ATM, on threonine 199 in *de novo* and naturally KSHV-infected cells. Phosphorylation occurs also in primary KS tumors at the same site. Additionally, v-Cyclin/CDK6 phosphorylation of NPM facilitates the interaction of LANA with the histone deacetylase HDAC1 to promote KSHV latency [114]. Indeed, NPM, is a HDAC recruiter involved in transcriptional repression during cell differentiation [115] and with viral inhibition of lytic gene expression [116]. Therefore, v-Cyclin is believed to function also as a modulator of the latent-lytic switch.

KSHV vFLIP activates the inhibitor of κB kinase (IKK) complex, triggering the direct activation of nuclear factor-κB (NF-κB) and the expression of anti-apoptotic genes and cytokine secretion [41,55]. vFLIP also negatively regulates autophagy in latently infected cells [56] and inhibits autophagy in KSHV-infected endothelial cells and lymphocytes [56]. Indeed, v-FLIP directly antagonizes the autophagy pathway limiting oncogene-induced and DDR-mediated senescence caused by KSHV [113]. 

Taken together, this evidence indicates that in infected cells KSHV stimulates uncontrolled cell proliferation evading the growth suppression induced by the DDR pathway, and that this may represent an “early dysregulation” which is vital in promoting tumorigenesis [114].

## 4. Activation of the DDR during KSHV Lytic Replication of KSHV

**Figure 3:**
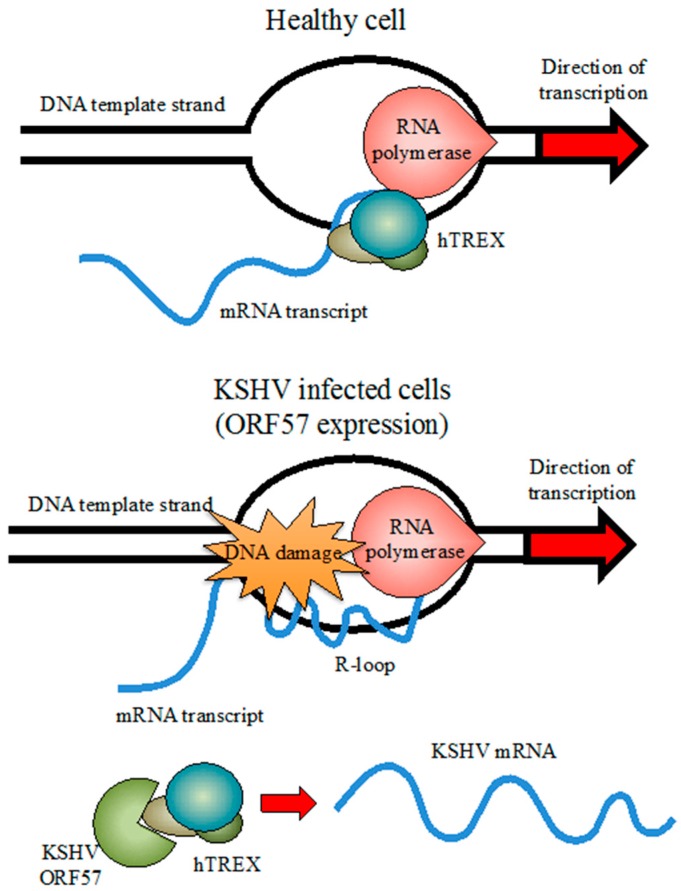
Genome instability and DNA damage induction by the KSHV ORF57 as proposed by Jackson *et al.* 2014 [118]. In a healthy cell, the evolutionarily conserved multiprotein complex hTREX plays a major role during biogenesis and stabilization of the newly transcribed mRNA.

Lytic reactivation in KSHV causes a severe and sustained DDR in infected cells as evidenced by high levels of γH2AX and these damages are induced before the viral DNA replication begins [83,117,118]. The induction of DSBs in cellular DNA may depend upon the activity of the viral mRNA export factor ORF57 [118]. Recent evidence has highlighted a possible link between mRNA export, genome instability, and cancer development [119]. The human Transcription and Export complex (hTREX) [120], has a central role in mRNA processing including the recruitment of the nuclear export receptor (TAP), to initiate and stabilize mRNA during transcription [121]. Aberrations in hTREX protein expression and function have been implicated in human cancer [119]. The KSHV ORF57 protein supports all stages of viral mRNA processing during lytic replication and promotes expression of KSHV genes [122]. At the same time, ORF57 enhances the splicing of several viral transcripts through an interaction with the cellular protein partner of Y14-Magoh (PYM) that recruits the cellular pre-initiation complex and enhances viral translation [122,123]. It was proposed that sequestration of hTREX by ORF57 could cause newly transcribed mRNA to form R-loops through annealing to the DNA template causing DSBs (Figure 3) [118].

In the presence of non-functional hTREX, caused by mutations or siRNA, the newly transcribed mRNA induces the production of unstable R-loops that cause DNA damage. Similarly, the expression of ORF57 during KSHV infection causes the sequestration of the hTREX complex, triggering genome instability.

RTA, encoded by ORF50, is the main protein mediating the switch between latency and lytic replication [124]. The expression of RTA and other immediate early genes precedes the expression of the delayed-early genes, which include a viral DNA polymerase, primase, helicase, and single-stranded binding proteins [37]. The last stage of lytic reactivation triggers late gene induction to produce structural proteins necessary for viral particle formation [7,124]. To promote an appropriate S-phase-like environment for replication and to induce the evasion of host cell defenses, KSHV needs to modulate the host cell cycle. KSHV evades host innate immunity by encoding several molecules with high homology to the host genes, including viral Bcl2, viral interleukin-6 (vIL-6), and four homologues of viral interferon regulatory factors (vIRF1-4) [125]. vIRF1 is encoded by ORF K9 and contains two domains, an N-terminal DNA binding domain (DBD) and C-terminal IRF interaction domain (IAD) [126,127]. The DBD shares 40% sequence similarity to the DBDs of human IRF3 and IRF7 and contains a helix-turn-helix (HTH) motif, which is common in IRFs and DNA binding proteins [127]. Deregulation of IRF3 and IRF7 mediated by vIRF1 induces disruption of cellular antiviral activity.

vIRF1 is a potent inhibitor of the histone acetyltransferase activity of p300. This process causes hypoacetylation of histones and alteration of the chromatin structure reducing expression of Interferons (IFNs), which are cytokines with the potential to inhibit cell growth and protect against viral infection [126,128]. Moreover, vIRF1 interact with and inactivate p53 by suppressing its acetylation and its transcriptional activation potential [129]. Specifically, vIRF1 interacts with the cellular p53 tumor suppressor at the level of the putative DNA binding region of vIRF1 and the central region of p53 [130]. As a consequence of the acetylation and transcriptional suppression of p53, vIRF1 prevents p53-mediated apoptosis, thus circumventing the host growth surveillance mechanism and facilitating an uncontrolled cell proliferation [130]. vIRF1 induces an increase in p53 ubiquitination through the deregulation of the Ubiquitin Specific Protease 7 (USP7) and the inhibition of the activating phosphorylation of S15 [131]. A further analysis revealed that vIRF1 interacts with ATM through its C-terminal domain reducing its activation, as shown by phosphorylation of S1981 [130]. vIRF also decreases the activation of downstream targets of ATM such as CHK2, H2AX, as well as p53, while the expression of the cell cycle regulatory protein CDC25 increased, supporting the notion that vIRF1 can suppress the downstream checkpoint activation by ATM [130]. 

A siRNA screening for novel regulators of KSHV reactivation identified MDM2, a repressor of p53, as a negative regulator of viral reactivation. Silencing of MDM2 promoted efficient activation of the viral lytic transcription program and viral reactivation. The activation of the lytic replication induced p53 response, DNA damage and a G2-phase arrest. Nevertheless, depletion of p21, which is a p53 target gene, restored cell cycle progression affecting virus reactivation and delaying the onset of virus replication [117]. Both p21 and CHK1, which are active during reactivation, are involved in the G2/M transition [117,132]. The early induction p21, which efficiently inhibit CDK1 in response to DNA damage, can cause the arrest of G2 and an increase in efficiency of KSHV reactivation [117,132]. The lytic protein encoded by ORF59 is a processivity factor (PF-8) for HHV-8 DNA polymerase Pol-8 (ORF9). PF-8 is a DNA-binding protein that associates both dsDNA and ssDNA independent of DNA sequence. When KSHV enters the lytic stage, PF-8 forms a replication complex together with other replication proteins encoded by KSHV supporting viral DNA synthesis and the subsequent production of infectious virus [133]. The pull-down assay and mass spectrometric analysis revealed that PF-8 interacts with KU70 and KU86. Hence, KSHV PF-8, by disrupting the interaction between the KU complex and DNA-PKcs, impairs NHEJ repair [134].

Overall, these results suggested that KSHV lytic replication induces DSBs, interferes with the DDR mechanism and can promote tumorigenesis [134].

## 5. Conclusions

After KSHV infection, a large number of molecules involved in DDR signaling undergo an extensive dysregulation. ATM, ATR, and DNA-PKcs as well as several downstream targets are modulated by KSHV during both latent infection and lytic replication, thus creating a favorable environment for virus persistence and viral replication [37]. In KS tumors, DDR is activated in early lesions [107]. The induction of the DDR pathway is generally associated with uncontrolled cellular proliferation. On the contrary, the downregulation of the DDR in advanced KS tumors is most probably due to the selection of mutations that allows cell survival [104].

Activation of the DDR signaling pathways by KSHV increases the sensitivity of virus-infected cells to DNA damaging agents, which promotes cell death [135]. Targeting protein components of the DDR pathway might thus offer a therapeutic opportunity to block the establishment of KSHV latent infection and, possibly treat KSHV-associated malignancies.

Indeed, several molecules have the potential to directly or indirectly modulate the DDR, most of which exert genotoxic effects. These include inhibitors of molecules involved in DNA synthesis, alkylation or covalent cross-linking of DNA strands that cause the cleavage of the sugar–phosphate backbone, as well as molecules that exert indirect roles in DNA replication [75].

Genotoxic agents that cause DNA damage, typically used in standard chemotherapeutic regimens, are also active for the treatment of KSHV-associated malignancies. Bleomycin, doxorubicin, and etoposide have been used as single agents or in combination for the treatment of KS. Administering chemotherapeutic agents such as doxorubicin and bleomycin together with vincristine, an alkaloid that promote apoptosis [136], have shown stronger responses compared to single agents [137]. The liposomal formulation of pegylated liposomal doxorubicin has a proven clinical efficacy, representing first-line treatment for patients with AIDS-associated Kaposi’s sarcoma [138].

Application of electrochemotherapy, which combines electroporation, delivered by short, intense electric pulses, with low doses of cytotoxic drugs, has been proven effective in the treatment of KS lesions [139]. The administration of electrochemotherapy and bleomycin to skin nodular lesions indicates that this approach might represent a suitable option for the treatment of KS skin nodules, given the reported complete remission of treated lesions in approximately 90% of patients, in the absence of relapses during a follow-up period of 6–48-months [140].

In addition, small-molecule inhibitors such as Nutlin-3 and RETRA, which disrupt the interaction of p53 and p73 with MDM2, can efficiently block KSHV-induced cell proliferation and cancer progression, promoting apoptotic cell death [141,142].

Other drugs can exert their activity by directly interacting with viral proteins that, in turn, modulate the DDR pathway. Particularly, glycyrrhizic acid, which inhibits the lytic replication of herpes viruses by reducing the expression of LANA, induces G1 cell cycle arrest and p53-mediated apoptosis [143].

Future studies focusing on the better understanding of the molecular mechanisms adopted by KSHV during latent and lytic replication and its ability to modulate the DDR will have important implications for designing novel therapeutic strategies to prevent and treat KSHV-associated malignancies.
